# SSeCKS/Gravin/AKAP12 attenuates expression of proliferative and angiogenic genes during suppression of v-Src-induced oncogenesis

**DOI:** 10.1186/1471-2407-6-105

**Published:** 2006-04-25

**Authors:** Yongzhong Liu, Lingqiu Gao, Irwin H Gelman

**Affiliations:** 1 Mucosal Immunology Unit, National Institutes of Health, Bethesda, MD 20892, USA; 2 Department of Cancer Genetics, Roswell Park Cancer Institute, Buffalo, NY 14263, USA

## Abstract

**Background:**

SSeCKS is a major protein kinase C substrate with kinase scaffolding and metastasis-suppressor activity whose expression is severely downregulated in Src- and Ras-transformed fibroblast and epithelial cells and in human prostate, breast, and gastric cancers. We previously used NIH3T3 cells with tetracycline-regulated SSeCKS expression plus a temperature-sensitive v-Src allele to show that SSeCKS re-expression inhibited parameters of v-Src-induced oncogenic growth without attenuating *in vivo *Src kinase activity.

**Methods:**

We use cDNA microarrays and semi-quantitative RT-PCR analysis to identify changes in gene expression correlating with i) SSeCKS expression in the absence of v-Src activity, ii) activation of v-Src activity alone, and iii) SSeCKS re-expression in the presence of active v-Src.

**Results:**

SSeCKS re-expression resulted in the attenuation of critical Src-induced proliferative and pro-angiogenic gene expression including Afp, Hif-1α, Cdc20a and Pdgfr-β, and conversely, SSeCKS induced several cell cycle regulatory genes such as Ptpn11, Gadd45a, Ptplad1, Cdkn2d (p19), and Rbbp7.

**Conclusion:**

Our data provide further evidence that SSeCKS can suppress Src-induced oncogenesis by modulating gene expression downstream of Src kinase activity.

## Background

SSeCKS, originally identified as a transcriptionally-suppressed gene in v-*src *and *ras*-transformed rodent fibroblast cells [1], is the orthologue of human GRAVIN/AKAP12 gene that encodes a kinase-scaffolding protein [2] that is targeted as an autoantigen in some cases of myasthenia gravis [3]. SSeCKS/Gravin/AKAP12 expression is severely downregulated in human prostate, breast and gastric cancer, partially relating to the mapping of the human gene to 6q24-25.1 [4], a cancer deletion hotspot [5]. Re-expression of SSeCKS to physiologic levels in Src- or Ras-transformed fibroblasts or epithelial prostate cancer cells suppresses morphological transformation, anchorage- and growth factor-independent proliferation, and metastatic potential, while restoring normal actin-based cytoskeletal architecture and cell-cycle controls on cyclin D1 expression [4,6,7]. SSeCKS also seems to control the blood-brain barrier by suppressing astrocyte-expressed vascular endothelial growth factor (VEGF) during the switch to normoxic conditions after birth [8]. A recent study indicates that the ability of SSeCKS to suppress lung metastasis formation by MatLyLu prostate cancer cells correlates with its suppression of VEGF 165 and 121 isoforms [9]. Interestingly, SSeCKS does not grossly alter the Src-mediated tyrosine phosphorylation of cellular substrates *in vivo *[6], strongly suggesting that SSeCKS suppresses tumorigenicity by re-establishing controls on downstream cytoskeletal and signaling pathways. However, it remains unclear which pathways are regulated by SSeCKS during tumor or metastasis suppression.

In this report, we analyzed how SSeCKS re-expression affects v-Src-induced oncogenic gene expression patterns using oligonucleotide microarrays and semi-quantitative RT-PCR techniques. Our data show that SSeCKS suppresses several critical proliferation- and angiogenesis-associated genes while it induces differentiation and cell cycle control functions, strongly suggesting that SSeCKS is capable of reprogramming normal gene expression controls downstream of activated Src.

## Methods

### Cells

S2-6 cells are NIH3T3 cells that encode a tetracycline (tet)-regulated tTA transactivator (Tet-OFF), S24 cells are S2-6 cells encoding a tet-regulated rat SSeCKS cDNA, and S24/ts72v-Src cells express temperature-sensitive v-Src whose kinase activity is only active at the permissive temperature (PT = 35°C), as described previously [6]. Cell cultures were maintained in complete DMEM supplemented with 10% calf serum, penicillin/streptomycin/amphotericin B, 2 μg/ml puromycin (S24 and S24/ts72v-Src cells), 65 μg/ml G418 (S24/ts72v-Src cells) and 0.7 mg/ml tet (Sigma).

### Oligonucleotide array analysis

1 μg of total RNA, isolated from comparable cell groups using TRIzol reagent (Invitrogen.), was reverse-transcribed into Cy-3- and Cy-5-labeled probes used to hybridize to Affymetrix A430 chips (Santa Clara, CA) according to the manufacturer's protocol. Fluorescence intensity for each chip was measured with an Affymetrix 428 Scanner. Data were derived from three independent microarray analyses performed for each cell type, and comparative analysis of resulting data was performed using software suites including GeneSpring v5.0 (Silicon Genetics), Data Mining Tool v3.0 (Affymetrix), GeneTraffic Uno (Iobion Informatics), dChip v1.1 (Harvard University) and SAM v1.15 (Stanford University) [10]. The mean hybridization signal for each sample was set as 1000 arbitrary units to normalize the signal values of all of the genes on the chip (global normalization) between different samples. The signal ratio of 2 or 0.5 was chosen as the criterion for induction or repression, respectively. In repeat experiments, most of the inter-experimental variation in gene expression (of the genes listed in Tables 3, 4, 5) was less than 2-fold, and only a few genes varied widely (e.g.- typically, 3.5- to 6-fold). However, these variations did not alter the trends in gene regulation (i.e.- up- or downregulation) by SSeCKS and/or v-Src.

### RT-PCR

1 μg of total RNA was primed with oligo-dT_16_, reverse transcribed into first strand cDNA and then amplified in the linear range using the SuperScript^® ^III RTS One-Step RT-PCR Kit (Invitrogen), as described previously [1], using the primer sets described in Table 1. The primer sets for Gpr56, Maff, Socs3, Afp, Ngfb, Gadd45a, Marcks, Hif1a, AFP, PDGFRB and HIF1A were purchased as QuantiTect Primer Assays (Qiagen). Optimization of RT-PCRs for semi-quantitative analysis was carried out after normalization using the β-actin mRNA as an internal control. PCR products were electrophoresed in 1.6% agarose gels, stained with ethidium bromide, and digitally imaged using a Chemi-Genius^2 ^BioImager (Syngene). Relative intensities of PCR bands were quantified using GeneTools image software analysis (Syngene).

### Western blot

Blotting analysis was performed as described previously [11] using the following primary antibodies at 1:1000 dilutions: PAb anti-SSeCKS [12], MAb anti- β-actin (Sigma), anti-Src [poY418] (BioSource International), MAb anti-v-Src (Ab-1; Oncogene Sciences). Secondary PAbs were either horseradish peroxidase-conjugated anti-rabbit or -mouse Ig (1:2,500; Chemicon) followed by Lumi-Light chemiluminescence reagent (Roche).

## Results and discussion

### Conditional expression of SSeCKS and v-Src activation in mouse fibroblast cell lines

**Figure 1:**
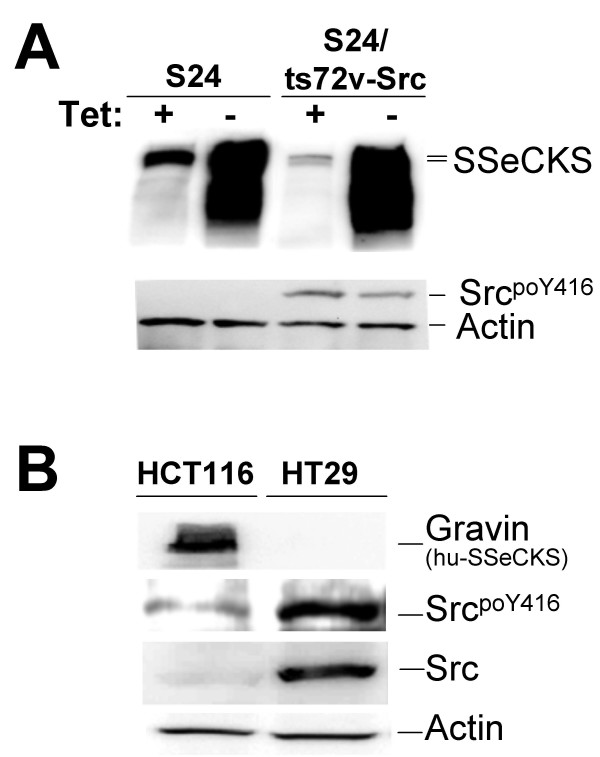
SSeCKS/Gravin/AKAP12 downregulation by activated Src. (**A**) Lysates from S24 and S24/ts72v-Src cells (grown at the PT) in the presence or absence of tet were immunoblotted for SSeCKS, activated Src (poY416) or actin. Note that SSeCKS overexpression (- tet) did not affect total ts72v-Src protein levels [6]. (**B**) A similar analysis as in panel A with lysates from HCT116 and HT29 human colon cancer cells with the addition of an immunoblot for total Src protein.

Our previous data indicated that SSeCKS overexpression in untransformed fibroblasts induces cell flattening as well as G1 phase growth arrest, the latter due to the suppression of growth factor-induced cyclin D1 expression [7]. The forced re-expression of SSeCKS could suppress v-Src-induced oncogenic growth *in vitro *without grossly affecting the ability of v-Src to phosphorylate cellular substrates [6]. Therefore, in order to identify genes differentially regulated by SSeCKS during G1 arrest or during suppression of Src-induced oncogenesis, we used NIH3T3-derived lines with tetracycline (Tet-OFF)-inducible SSeCKS expression ("S24 cells") as well as S24 cells expressing a temperature-sensitive v-Src allele ("S24/ts72v-Src"). The ectopic expression of SSeCKS was strongly induced (>25-fold) in the absence of tetracycline (tet), yet in the presence of tet, ts72v-Src activation (35°C) resulted in a >10-fold decrease in endogenous SSeCKS levels (Fig. 1A), confirming our initial identification of SSeCKS as a Src-suppressed gene [1]. Moreover, SSeCKS overexpression did not affect Src autophosphorylation activity, as monitored by phospho-Y416 levels (Fig. 1A). The relationship between increased Src activity levels and decreased SSeCKS levels is also maintained when comparing the metastatic human colon cancer cell line, HT29 (high Src activity, low Gravin), with the low metastatic line, HCT116 (low Src activity, high Gravin) (Figure 1B).

We previously showed that growth of S24/ts72v-Src cells in 0.04 μg/ml tet resulted in re-expression of ectopic SSeCKS to the physiologic levels found in parental NIH3T3 cells [6]. Therefore, as described in Table 2, we compared differential gene expression for the following conditions: i) SSeCKS overexpression in untransformed cells (S24 grown in + vs. – tet at 37°C), ii) v-Src activation without SSeCKS re-expression ([S24/ts72v-Src grown in the presence of tet at 35°C vs. 39.5°C] minus [S24 grown in the presence of tet at 35°C]), or iii) SSeCKS re-expression in the presence of activated v-Src (S24/ts72v-Src at 35°C grown in + vs. – tet).

### Profile of SSeCKS-regulated gene expression

We identified 45 genes whose expression was altered 2-fold or more by SSeCKS overexpression using at least three independent Affymetrix microarray analyses, as described in Materials and Methods, of RNA from S24 cells grown at 37°C in the presence or absence of tet. We then subtracted gene expression effects induced by the tTA transactivator alone in the absence of SSeCKS expression, that is, RNA derived from S2-6 parental cells [13] grown in the absence of tet. Given our prior results that SSeCKS overexpression inhibits G1->S transition in untransformed fibroblasts [7], it was not unexpected that ectopic SSeCKS could induce tumor suppressor/cell cycle negative control genes such as those encoding Rb, p19^INK4 ^or Rassf5, while suppressing cell cycle promoting genes, such as cyclins D, A, B and F, Fos-like antigen 1, Afp [14] and the CDC20 homolog, or neoplasia-promoting genes such as Hmgb3 [15] or Cebpb [16] (Table 3). Similarly, SSeCKS induction of Tgf-α and -β, and of Ngf-β, and the downregulation of Mtap7, is consistent with the pseudo-differentiated, non-polar phenotype displayed during SSeCKS-induced cell flattening [13]. The loss of PDGFR-β expression, which we have described elsewhere [17], likely relates to the ability of SSeCKS to suppress angiogenesis in the brain [8] and in tumor neovasculature. Of the signal transduction genes, the upregulation of Ptplad1, Traf6 [18] and Lmo4 [19] were consistent with the ability of SSeCKS to control cell cycle progression. Stam2 upregulation or Peg3 downregulation may facilitate SSeCKS-mediated anti-apoptosis based on reported roles for Stam2 role T cell survival signaling [20] and for Peg3 in neuronal death [21]. In contrast, SSeCKS unexpectedly upregulated several genes such as Gpr56, Prkce and Btbd2 which have been reported to be upregulated in human cancers [22,23] or involved in inducing cell cycle progression [24], whereas it downregulated several genes such as Nasp, known to antagonize cell cycle progression [25]. The role of other genes, such as Gng2, in SSeCKS regulation remain unclear although they are typically involved in specialized neuronal signaling responses [26]; upregulation might suggest that SSeCKS induces signaling pathways more associated with differentiated, especially neuronal, cells. Interestingly, we previously published that SSeCKS overexpression in S24 cells induces long axonal-like extensions which, when stained for SSeCKS, exhibit periodic, pearl-like enrichments of SSeCKS that are strikingly similar to SSeCKS-enriched structures in developing hippocampal axons [27]. The severe downregulation of Dusp9- which normally antagonizes insulin-mediated adipogenesis [28]- may reflect an ability by SSeCKS to induce glucose uptake and/or metabolism, though this has not been studied. Mycbp is known to inhibit PKA activation at various cell sites by preventing recruitment of the PKA catalytic subunit to the scaffolding proteins, S-AKAP84 and AKAP95 [29]. Thus, Mycbp upregulation by SSeCKS- also a known AKAP [2]-may reflect a higher level of temporal and/or spatial control of PKA activity by SSeCKS.

### Profile of v-Src-regulated gene expression

24 genes were identified whose expression varied 2-fold or more by v-Src activation. Specifically, we compared microarray data from RNAs derived from S24/ts72v-Src cells grown in the presence of tet (i.e.- no ectopic SSeCKS) at either 35°C (the PT for Src kinase activity) or 39.5°C (the NPT) (Table 4, column A). To identify possible gene changes induced by incubation at 39.5°C (and not by the loss of Src activity), we compared microarray data derived from S24/ts72v-Src cells versus S24 cells grown in the presence of tet at 35°C (Table 4, column B). However, gene expression changes that occur under the latter conditions only (e.g.- Sh3bp5) might also reflect *bona fide *v-Src-induced changes that are negated at the NPT, and thus, we included both column A and B data in our overall analysis.

Six of the 24 genes (25%) were identified in Table 3 as being inversely regulated by SSeCKS, i.e.- if they were induced by SSeCKS, they were suppressed by v-Src, or vice-a-versa. These include Peg3, Fosl1, Hmgb3, Gfra1, Il1rl1 and Afp. In contrast, Igfbp4 is dowregulated both by SSeCKS overexpression and v-Src activation, lessening confidence that this gene plays a significant role in oncogenesis.

Rgs16, Id2 and Socs3 have paradoxical roles in that they play both positive and negative functions in cancer. For example, Rgs16- whose expression is induced by v-Src in our system- is a GTPase activating protein for Gα_i _[30], and Gα_i _is known to cooperate with Src in oncogenic transformation [31]. However, Rgs16 activity is required for retinoid-induced growth inhibition [32], suggesting that it could antagonize cancer progression. Similarly, Socs3- whose expression is also induced by v-Src, is known to inhibit insulin-mediated proliferation signaling [33] and to be downregulated in head and neck squamous cell carcinomas [34], yet it is upregulated in breast cancer [35]. Id2 plays positive roles in cancer, possibly by inhibiting parameters of differentiation [36], yet decreased Id2 levels correlate with increased invasive potential in human breast cancer cases [37].

The increased levels of the transcription factors Maff, Runx1 and Etv4, and the decreased levels of Centd3, Id4, Spon2, and Cbr2 by v-Src seem to directly relate to cancer progression. Specifically, Maff is overexpressed in some forms of cancer [38] and Maf proteins play direct roles in controlling v-Src-regulated gene expression [39]. Runx1 induces cell cycle progression [40] and increased Runx1 levels are found in chronic myelogenous leukemias [41]. Etv4 levels are increase in gastric cancers [42]. Centd2 is a GAP for RhoA whose activity is blocked by phosphorylation by Src-family kinases [43]. Id4 is downregulated in colon [44] and gastric cancers [45]. Spon2 and Cbr2 levels are decreased significantly in lung cancers [46,47]. In contrast, v-Src induction of Prkg2 does not make apparent sense given that higher Prkg2 levels correlate with increased patient survival rates for lung cancer [48]. Finally, it is unclear how the differential regulation of Sh3bp, Atoh6, Tcfec and Pscdbp would affect v-Src-induced oncogenesis.

### Profile of gene expression in cells expressing activated Src plus physiologic SSeCKS levels

46 genes were identified whose expression was altered under conditions of activated v-Src and SSeCKS to physiologic levels (Table 5). 16 of these genes (36%) were identified in screens described in Tables 3 or 4. Of these, 12 genes (Gpr56, Gng2, Ptplad1, Marcks, Cdkn2d, Cdc2a, Hmgb3, Ngfb, Gfra1, Il1rl1, Pdgfrb and Afp) exhibited the same expression control (i.e.- up-or downregulation) as detected after SSeCKS overexpression alone (Table 3). This strongly suggests that these genes are markers for SSeCKS-mediated growth arrest and/or tumor suppression. The remaining 4 pre-identified genes (Socs3, Maff, Id2 and Atoh6) are regulated in the same manner as in v-Src-transformed cells (Table 4) and thus, it is likely that these genes remain controlled by v-Src yet are not sufficient for the oncogenic phenotype.

SSeCKS reverses the expression of several genes that might either antagonize v-Src-induced oncogenesis or function as markers for non-transformed cells. Specifically, SSeCKS induces the expression of Gadd45a- a gene known to inhibit progression at either S or G2/M phases [49] or to induce density-dependent G1 phase arrest [50], Cdkn2d, Rbbp7- which inhibits mitogen- and oncogene-induced c-Fos activation [51], and Dub1- whose overexpression induces G1 arrest [52]. In the same context, SSeCKS suppresses the expression of Map2kb- whose activity is increased in Ras-mediated invasiveness and metastatic potential [53], Rgs2- whose increased expression in mantle cell lymphomas correlates with increased metastatic potential [54], Tcfap4- a regulator of caspase-9 mediated apoptosis [55], Cdc2a, Hif1a- a mediator of tumor angiogenesis induced by v-Src [56,57] and activated c-Src [58,59], Ddit4- an Hif1a-inducible gene [60], and Pdgfrb. Interestingly, SSeCKS re-expression may facilitate increased immune surveillance of tumor cells by the induction of Hsp4a, a tumor antigen carrier that increases the immunogenicity of colon cancer cells in a murine model [61]. Although the decrease in Caspase-7 (Casp7) expression correlates with the decreased apoptotic index of v-Src cells re-expressing SSeCKS [6], it is unclear how the concomitant increase in Stk4- a pro-apoptotic serine/threonine kinase [62], affects cell survival after SSeCKS re-expression.

A set of genes seems to remain regulated by v-Src during SSeCKS re-expression, suggesting that their expression levels are not sufficient to induce or maintain oncogenic transformation in the presence of physiologic levels of SSeCKS. These include Ptpn11- which can activate Src-family kinases [63] and which is required for v-Src-induced morphological transformation [64], Pip5k1b- whose product is activated by Rho-family GTPases in cancer cells [65], Srpk2- a gene upregulated after retinoid-induced differentiation of HL60 leukemia cells [66], Dusp10- a negative regulator of MAP kinases and antagonist of prostate cancer cell proliferation [67], Marcks, Timp3- a gene downregulated in squamous cell carcinomas and prostate adenocarcinomas [68,69], Gdf5- a growth arrest inducer in mouse B cells [70], and Gas1- a gene downregulated in EGF-induced hepatocellular carcinoma [71] and in Ras-transformed cells [72]. The involvement of S100a8 is unclear because although its expression is induced in prostate cancer [73], its expression is downregulated in the metastatic breast cancer cell line, MDA-MB-231, compared to the non-metastatic MCF-7 [74].

**Figure 2:**
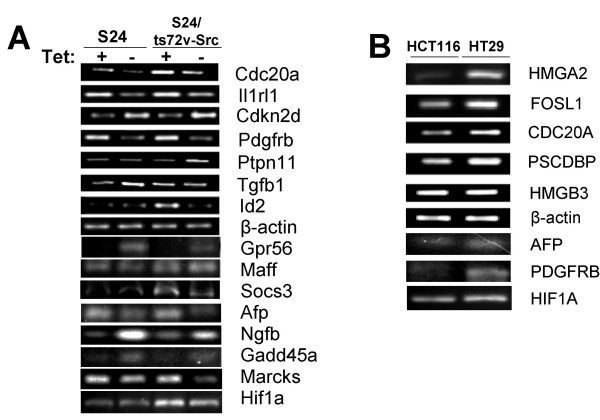
Verification of differential gene expression using semi-quantitative RT-PCR. Equal amounts of total RNA isolated from S24 or S24/ts72v-Src cells (grown at the PT) in the presence or absence of tet (Panel A) or from HCT116 or HT29 (Panel B) were subjected to RT-PCR analysis as described in Materials and Methods using primer sets described in Table 1. These results are typical of at least two independent experiments.

In order to verify the gene expression changes identified in the microarray studies, several genes were assayed by semi-quantitative RT-PCR using primer sets shown in Table 1. Thus, RNAs derived from S24 or S24/ts72v-Src cells grown at the PT in the presence or absence of tet were amplified by RT-PCR as described in Materials and Methods and then the electrophoresed products were quantified by digital densitometry. Fig. 2A and Table 6 show a strong concordance between the regulation of Cdc2a, Il1rl1, Cdkn2d, Pdgfrb, Ptpn11, Tgfb1, Id2, Gpr56, Maff, Socs, Afp, Ngfb, Gadd45a, Marcks and Hif1a by either SSeCKS alone or SSeCKS re-expression in the presence of v-Src as gauged by both the microarray and RT-PCR assays. This strengthens the notion that the genes identified by our microarray comparisons reflect *bona fide *examples of differential expression due to SSeCKS overexpression or SSeCKS re-expression in the presence of active v-Src. Finally, we performed semi-quantitative RT-PCR on several genes shown to be induced by active v-Src (Table 3) using RNAs from HT29 and HCT116, representing cells with high and low levels of c-Src activity, respectively (Fig. 1B). Fig. 2B shows significantly higher levels of HMGA2, FOSL1, CDC20A, PSCDBP, AFP, PDGFRB and HIF1A expression in HT29 cells, which correlates with the v-Src-induced levels found for the mouse orthologues in Table 4 (for both cell comparisons A and B). In contrast, HMGB3 transcript levels were similar in HT29 and HCT116 cells, correlating with the finding that its mouse orthologue was likely not induced by v-Src (Table 4, column B). Taken together, these data strongly suggest that activated Src and SSeCKS/Gravin control similar sets of genes in both human and mouse fibroblasts and epithelial cells.

## Conclusion

We have identified sets of genes whose expression is controlled either by SSeCKS or v-Src alone, or only after re-expression of SSeCKS in the presence of activated v-Src. Our data suggest that some or all of the SSeCKS-regulated genes may either directly antagonize v-Src-induced oncogenesis or may serve as markers for non-oncogenic cells. Moreover, our analysis has identified a set of genes previously thought to contribute to the oncogenic phenotype yet which are insufficient to induce oncogenesis in the presence of physiologic levels of SSeCKS. In sum, our data indicate that SSeCKS may antagonize Src-induced oncogenesis through the normalization of functions controlling mitogenic signaling pathways, cell cycle progression, transcriptional regulation and apoptosis.

## Abbreviations

SSeCKS, Src Suppressed C Kinase Substrate; PKC, protein kinase C; RT-PCR, reverse transcription-polymerase chain reaction; VEGF, vascular endothelial growth factor; PT, permissive temperature; NPT, non-permissive temperature; PAb, polyclonal antibody; MAb, monoclonal antibody; Ig, immunoglobulin; tet, tetracycline.

## Competing interests

The author(s) declare that they have no competing interests.

## Authors' contributions

YL performed the work as a postdoctoral fellow in the lab of IHG and thus, both contributed to the conceptual framework of this study. Both YL and IHG produced and approved the final written manuscript. LG performed many of the semi-quantitative RT-PCR assays.

## Pre-publication history

The pre-publication history for this paper can be accessed here:



## Figures and Tables

**Table 1:** Summary of primer sequences and positions

**Target gene **	**Sequence of forward and reverse primers **	**position**
**Mouse**		
Pdgfr-β	5'-AGCTACATGGCCCCTTATGA-3'	2749–2768
	5'-GGATCCCAAAAGACCAGACA-3'	3069–3115
Tgfβ-1	5'-CGGGGCGACCTGGGCA CCATCCATGAC-3'	1597–1623
	5'-CTGCTCCACCTTGGGCTTGCGACCCAC-3'	1975–2001
Cdc20	5'-GCTGGTTCTGGTGACATCCT-3'	757–776
	5'-TGTTCCAACTGAGGGAGCTT-3'	939–958
Ptpn11	5'-AGTCCAAAGTGACCCACGTC-3'	623–643
	5'-AGCGTCTCAAACTCTTCCCA-3'	874–893
Id2	5'-GGACATCAGCATCCTGTCCT-3'	383–402
	5'-AACGGTATCACAGTCCAGGC-3'	659–678
Il1rl1	5'-CGCTCGACTTATCCTGTGGA-3'	299–318
	5'-AGCTTGGCGGCTTTTTATGT-3'	490–509
Cdkn2d	5'AGCTTGGCGGCTTTTTATGT-3'	212–229
	5'-CGGTCCCATTACTTGTCAC-3'	460–442
β- actin	5'-TTCTTTGCAGCTCCTTCGTTGCCG-3'	33–56
	5'-TGGATGGCTACGTACATGGCTGGG-3'	467–490
Gpr56	Qiagen QuantiTect Assay, cat. #QT00178689	
Maff	Qiagen QuantiTect Assay, cat. #QT00133224	
Ngfb	Qiagen QuantiTect Assay, cat. #QT00093464	
Socs3	Qiagen QuantiTect Assay, cat. #QT00100331	
Gadd45a	Qiagen QuantiTect Assay, cat. #QT00249655	
Marcks	Qiagen QuantiTect Assay, cat. #QT00252973	
Hif1a	Qiagen QuantiTect Assay, cat. #QT00182532	
Afp	Qiagen QuantiTect Assay, cat. #QT00174020	
		
**Human**		
CDC20A	5'-GGGTTCCTCTGCAGACATTC-3'	1034–1053
	5'-TGTAATGGGGAGACCAGAGG-3'	1215–1234
PSCDBP	5'-TCAATGCAGCAATTGGAGTC-3'	741–761
	5'-ATGTCAATGCACGTCAGCAT-3'	920–940
FOSL1	5'-CCAAGCATCAACACCATGAG-3'	302–321
	5'-GGGCTGATCTGTTCACAAGG-3'	473–492
HMGA2	5'-CGAAAGGTGCTGGGCAGCTCCGG-3'	786–808
	5'-CCATTTCCTAGGTCTGCCTCTTG-3'	1086–1110
HMGB3	5'-ACAACCGAGACAAACCCTTG-3'	1107–1126
	5'-CCCCTTTGTCCACAGCTAAG-3'	1290–1310
β- ACTIN	5'-GCTCGTCGTCGACAACGGCTC-3'	93–113
	5'-CAAACATGATCTGGGTCATCTTCTC-3'	445–421
AFP	Qiagen QuantiTect Assay, cat. #QT00085183	
PDGFRB	Qiagen QuantiTect Assay, cat. #QT00082327	
HIF1A	Qiagen QuantiTect Assay, cat. #QT00083644	

**Table 2:** The cell groups/conditions used to compare gene expression

Cells/Growth condition	Comparison
NIH3T3/S24 cells, + vs. – tet, 37°C	SSeCKS overexpression
([S24/ts72v-Src cells, 35°C vs. 39.5°C] minus S24 cells, 35°C), + tet (1 μg/ml)	v-Src activation
S24/ts72v-Src cells, 35°C, 1.0 vs.0.04 μg/ml tet	SSeCKS re-expression plus v-Src activation

**Table 3:** Genes Regulated by SSeCKS in NIH3T3 fibroblasts^a ^A value ≥ 2 represents induction; a value ≤ 0.5 represents repression

**Gene**	Symbol	**Modulation**
**Signal transduction**		
*G protein-coupled receptor 56*	Gpr56	126.24*
*BTB (POZ) domain containing 2*	Btbd2	10.85
*Guanine nucleotide binding protein (G protein), gamma 2 subunit*	Gng2	9.58*
*Ras association (RalGDS/AF-6) domain family 5*	Rassf5	8.75
*Calcium binding protein 2*	Cabp2	8.11
*Butyrate-induced transcript 1*	Ptplad1	4.08*
*Signal-transducing adaptor protein-2*	Stam2	3.20
*Protein kinase C, epsilon*	Prkce	2.36
*Interferon-stimulated protein*	G1p2	2.30
*Tnf receptor-associated factor 6*	Traf6	2.23
*RAB33B, member of RAS oncogene family*	Rab33b	2.17
*Insulin-like growth factor 2, binding protein 3*	Igf2bp3	0.49
*Myristoylated alanine rich protein kinase C substrate*	Marcks	0.48*
*Insulin-like growth factor binding protein 4*	Igfbp4	0.47
*N-myc downstream regulated 1*	Ndrg1	0.41
*Paternally expressed 3*	Peg3	0.27
*Dual specificity phosphatase 9*	Dusp9	0.15
**Cell cycle and transcriptional regulator**		
*Retinoblastoma 1*	Rb1	3.48
*c-myc binding protein*	Mycbp	3.41
*Histone 2, H2aa1*	Hist2h2aa1	3.07
*Histone 3, H2a*	Hist3h2a	2.99
*Zinc fingers and homeoboxes protein 1*	Zhx1	2.79
*Cyclin-dependent kinase inhibitor 2D (p19, inhibits CDK4)*	Cdkn2d	2.73*
*CCAAT/enhancer binding protein (C/EBP), beta*	Cebpb	0.50
*Cyclin F*	Ccnf	0.48
*Cyclin A2*	Ccna2	0.47
*High mobility group box 1*	Hmgb1	0.46
*cell division cycle 20 homolog (S. cerevisiae)*	Cdc20a	0.46*
*Cyclin B1*	Ccnb1	0.44
*LIM domain only 4*	Lmo4	0.44
*Fos-like antigen 1*	Fosl1	0.41
*Cyclin D1*	Ccnd1	0.37
*High mobility group box 3*	Hmgb3	0.24*
*Nuclear autoantigenic sperm protein (histone-binding)*	Nasp	0.06
**Cytoskeleton**		
*Synaptonemal complex protein 3*	Sycp3	8.34
*Microtubule-associated protein 7*	Mtap7	0.18
**Growth factor and receptor**		
*Transforming growth factor, beta 1*	Tgfb1	2.33
*Nerve growth factor, beta*	Ngfb	2.28*
*Transforming growth factor alpha*	Tgfa	*2.27*
*Platelet derived growth factor receptor, beta polypeptide*	Pdgfrb	0.30*
*Interleukin 1 receptor-like 1*	Il1rl1	0.29*
*Glial cell line derived neurotrophic factor family receptor alpha 1*	Gfra1	0.15*
**Others**		
*Alpha fetoprotein*	Afp	0.32*

^a^S24 MEF, 37°C, Tet (-) vs (+)

* Similar effects after SSeCKS re-expression in v-Src transformed cells (Table 5).

**Table 4:** Genes regulated by ts72v-Src activation A value ≥ 2 represents induction; a value ≤ 0.5 represents repression

		**Modulation**	
		
**Gene**	***Symbol***	**A**	**B**
**Signal Transduction**			
*regulator of G-protein signaling 16*	Rgs16	10.13	12.70
*suppressor of cytokine signaling 3*	Socs3	3.89	3.00
*paternally expressed 3*	Peg3	3.22*	3.50*
*protein kinase, cGMP-dependent, type II*	Prkg2	2.53	2.30
*insulin-like growth factor binding protein 4*	Igfbp4	0.15**	0.42**
*SH3-domain binding protein 5 (BTK-associated)*	Sh3bp5	NC	0.29
*ARF-GAP, RHO-GAP, ankyrin repeat and plekstrin homology domains-containing protein 3*	Centd3	0.08	0.05
			
**Cell cycle and transcriptional regulator**			
*v-maf musculoaponeurotic fibrosarcoma oncogene family, protein F*	Maff	17.03	3.00
*high mobility group AT-hook 2*	Hmga2	6.96	5.00
*runt related transcription factor 1, RUN X1/AML-1*	Runx1	6.77	5.50
*ets variant gene 4 (E1A enhancer binding protein, E1AF)*	Etv4	6.68	8.30
*fos-like antigen 1*	Fosl1	4.47*	2.80*
*high mobility group box 3*	Hmgb3	3.73*	3.30*
*transcription factor EC*	Tcfec	NC	63.00
*inhibitor of DNA binding 4*	Id4	0.22	NC
*inhibitor of DNA binding 2*	Id2	NC	0.43
*basic helix-loop-helix transcription factor 6*	Atoh8	0.15	0.29
			
**Growth factor and receptor**			
*glial cell line derived neurotrophic factor family receptor alpha 1*	Gfra1	4.17*	7.00*
*interleukin 1 receptor-like 1*	Il1rl1	4.20*	3.50*
			
**Others**			
*pleckstrin homology, Sec7 and coiled-coil domains, binding protein*	Pscdbp	4.08	3.30
*alpha fetoprotein*	Afp	3.89*	3.80*
*spondin 2, extracellular matrix protein*	Spon2	0.13	0.23
*carbonyl reductase 2*	Cbr2	0.11	0.07

A, S24/ts72v-Src cells, 35°C vs 39.5°C, + tet.

B, S24/ts72v-Src cells, 35°C vs S24 MEF 35°C, + tet.

*, opposite effect when compared to SSeCKS overexpression alone (Table 3).

**, similar effects when compared to SSeCKS overexpression alone (Table 3).

NC, no change.

**Table 5:** Genes regulated by SSeCKS in v-Src transformed cells^a ^A value ≥ 2 represents induction; a value ≤ 0.5 represents repression

**Gene **	Symbol	**Modulation**
**Signal transduction**		
*Cytokine inducible kinase*	Plk3	52.35
*G protein-coupled receptor 56*	Gpr56	49.00*
*Guanine nucleotide binding protein (G protein), gamma 2 subunit*	Gng2	7.16*
*Protein tyrosine phosphatase, non-receptor type 11*	Ptpn11	5.86
*Serine/threonine kinase 4*	Stk4	3.97
*RAB20, member RAS oncogene family*	Rab20	3.92
*Phosphatidylinositol-4-phosphate 5-kinase, type 1 beta*	Pip5k1b	3.50
*Growth arrest and DNA-damage-inducible 45 alpha*	Gadd45a	3.43
*Butyrate-induced transcript 1*	Ptplad1	3.29*
*Suppressor of cytokine signaling 3*	Socs3	2.07***
*Serine/arginine-rich protein specific kinase 2*	Srpk2	0.45
*Mitogen activated protein kinase kinase 6*	Map2k6	0.44
*Regulator of G-protein signaling 2*	Rgs2	0.38
*Ras and Rab interactor 2*	Rin2	0.33
*Dual specificity phosphatase 10*	Dusp10	0.17
*Myristoylated alanine rich protein kinase C substrate*	Marcks	0.12*
		
**Cell cycle and transcriptional regulator**		
*Histone 1, H2bp*	Hist1h2bp	3.61
*Cyclin-dependent kinase inhibitor 2D (p19, inhibits CDK4)*	Cdkn2d	2.46*
*Retinoblastoma binding protein 7*	Rbbp7	2.39
*Histone 2, H3c2*	Hist2h3c2	2.28
*v-maf musculoaponeurotic fibrosarcoma oncogene family, protein F*	Maff	2.28***
*Inhibitor of DNA binding 2*	Id2	0.44***
*Transcription factor AP-4 (activating enhancer-binding protein 4)*	Tcfap4	0.26
*Cell division cycle 20 homolog (S. cerevisiae)*	Cdc2a	0.25*
*Basic helix-loop-helix transcription factor 6*	Atoh6	0.24***
*High mobility group box 3*	Hmgb3	0.24*^/^**
*Hypoxia inducible factor 1, alpha subunit*	Hif1a	0.22
*Kruppel-like factor 7 (ubiquitous)*	Klf7	0.10
		
**Growth factor and receptor**		
*Chemokine (C-C motif) ligand 2*	Ccl2	4.26
*Nerve growth factor, beta*	Ngfb	2.38*
*Glial cell line derived neurotrophic factor family receptor alpha 1*	Gfra1	0.33*^/^**
*Interleukin 1 receptor-like 1*	Il1rl1	0.23*^/^**
*Platelet derived growth factor receptor, beta polypeptide*	Pdgfrb	0.18*
*Transforming growth factor, beta 2*	Tgfb2	0.14
		
**Others**		
*S100 calcium binding protein A8 (calgranulin A)*	S100a8	16.22
*Fatty acid desaturase 3*	Fads3	8.00
*Heat shock protein 4*	Hspa4	4.29
*Deubiquitinating enzyme 1*	Dub1	3.23
*alpha fetoprotein*	Afp	0.50*^/^**
*Tissue inhibitor of metalloproteinase 3*	Timp3	0.33
*HIF-1 responsive RTP801*	Ddit4	0.26
*5-azacytidine induced gene 1*	Azi1	0.24
*Growth differentiation factor 5*	Gdf5	0.18
*Growth arrest specific 1*	Gas1	0.18
*Caspase 7*	Casp7	0.18
*retinitis pigmentosa 1 homolog (human)*	Rp1h	0.12
*protocadherin 18*	Pcdh18	0.07

^a^, S24/ts72v-Src MEF, 35 °C, + vs – tet.

*, similar effects when compared to SSeCKS overexpression alone (Table 3).

**, opposite effects when compared to v-Src activation (Table 4).

***, similar effects when compared to v-Src activation (Table 4).

**Table 6:** Comparison of microarray and RT-PCR data

	**Fold change (array)**		**Fold change (RT-PCR)**^a^	
		
Gene	SSeCKS Induction^b^	SSeCKS induction plus v-Src activation^c^	SSeCKS induction	SSeCKS induction plus v-Src activation
Cdc2a	0.46	0.25	0.27	0.25
Il1rl1	0.29	0.23	0.31	0.36
Cdkn2d	2.73	2.46	3.10	3.50
Pdgfrb	0.30	0.18	0.34	0.30
Ptpn11	NC*	5.86	NC	3.04
Tgfb1	2.33	NC	2.50	NC
Id2	NC	0.44	NC	0.33
Gpr56	126.24	49.00	45.0	28.0
Maff	NC	2.28	NC	3.5
Socs3	NC	2.07	NC	2.4
Afp	0.32	0.50	0.4	0.2
Ngfb	2.28	2.38	8.0	2.5
Gadd45a	NC	3.43	4.0	3.0
Marcks	0.48	0.12	NC	0.2
Hif1a	NC	0.22	NC	0.3

^a^, based on two independent experiments. SE < 0.005.

^b^, from Table 3.

^c^, from Table 5.

NC, no change.
